# Contributing factors to health‐related quality of life in multiple sclerosis

**DOI:** 10.1002/brb3.1466

**Published:** 2019-11-11

**Authors:** Tamás Biernacki, Dániel Sandi, Zsigmond Tamás Kincses, Judit Füvesi, Csilla Rózsa, Klotild Mátyás, László Vécsei, Krisztina Bencsik

**Affiliations:** ^1^ Department of Neurology Faculty of General Medicine Albert Szent‐Györgyi Clinical Centre University of Szeged Szeged Hungary; ^2^ Jahn Ferenc Dél‐Pest Hospital Budapest Hungary; ^3^ Markhot Ferenc Teaching Hospital Eger Hungary; ^4^ MTA – SZTE Neuroscience Research Group Szeged Hungary

**Keywords:** cognitive impairment, depression, fatigue, multiple sclerosis, quality of life, sex differences

## Abstract

**Background:**

Health‐related quality of life (HRQoL) is lower in people with multiple sclerosis (PwMS) compared to the healthy population, psychological symptoms accompanying multiple sclerosis (MS) have a serious impact on the HRQoL of PwMS. Data regarding the subject, however, remain conflicting.

**Objectives:**

To evaluate the patients' sociodemographic attributes, education, fatigue, depression, and cognitive impairment level of impact on the HRQoL for the whole cohort as well as comparing the sexes.

**Materials and Methods:**

Three hundred and twenty‐two relapse‐remitting MS patients filled out the Fatigue Impact Scale (FIS), Beck Depression Inventory (BDI), MS Quality of Life‐54 (MSQoL‐54) questionnaires, cognitive impairment were identified using Brief International Cognitive Assessment for MS (BICAMS) test. The patients' data were acquired from our clinic's MS registry or from patients' files.

**Results:**

Depression and fatigue were found to have the most ubiquitous and robust effect on the overall and any given subdivision of the HRQoL composite. Other factors had a slight effect on some of the subscales when the whole cohort was evaluated. When the genders were compared, differences were found on 10 domains.

**Conclusion:**

Psychopathological symptoms have a more powerful influence on the HRQoL of MS patients than physical impairment, also these symptoms influence men's and women's HRQoL with different power. This invokes the need for complex and personalized care in the treatment of PwMS. Ours is the first study to show a difference between the sexes in this regard.

## Graphical abstract

The determining factors of the health‐related quality of life of MS patients were assessed. Psychopathological, not physical symptoms were found to be the main influencers of one's health‐related quality of life (HRQoL). Also a difference between men and women was found regarding which factors determine a patient's HRQoL and the power of influence a given factor has on one's HRQoL.

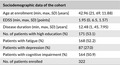

## 1 INTRODUCTION

Multiple sclerosis (MS) is one of the most common debilitating, progressive neuroinflammatory disorders. Most often affects young and middle‐aged adults in their most productive lifetime increasing the enormous financial burden of the disease (Confavreux, Aimard, & Devic, 1980; Zsiros et al., 2014). It affects patients' life in many aspects. As the disease progresses, some of the exacerbations heal with residual symptoms, thus people with MS (PwMS) are forced to live with more and more impairments and restrictions to their everyday lifestyle. As expected, several studies have shown the health‐related quality of life (HRQoL) to be affected in PwMS compared with the general population. However, when compared to other autoimmune conditions (namely rheumatoid arthritis and inflammatory bowel disease), PwMS have the lowest perceived HRQoL (Rudick, Miller, Clough, Gragg, & Farmer, 1992). Various factors have been identified over the years by several authors to have an influence on the HRQoL. Lower household income, higher EDSS score, lower score on the 9‐hole‐peg test, weaker coping capacity, and a more debilitating, more progressive disease course have been all linked to a lower HRQoL, and lower HRQoL has been shown to foreshadow worse survival in PwMS (Ruet et al., 2013; Lanzillo et al., 2016; Kurtzke, 1983; Solaro, Gamberini, & Masuccio, 2018).

In the past two decades, it was revealed that most PwMS have cognitive and psychological symptoms as well as physical ones and that these symptoms can develop at any stage of the disease (Glanz et al., 2007; Amato et al., 2012). The lifetime prevalence of depression in PwMS is about 50%; 43%–70% suffer from cognitive impairment (CI) and fatigue turned out to be the most common symptom of the disease, with a prevalence rate over 90% (Ben Ari Shevil, Johansson, Ytterberg, & von Bergstrom, 2014; Chiaravalloti & DeLuca, 2008). In recent years, several evaluations concluded that these psychological symptoms have a serious impact on the HRQoL independently from the patients' physical state. CI was shown to be one of the most crucial factors in patients becoming unemployed and leading to several other serious burdens (Langdon, 2011). Fatigue and depression were both shown to be individual factors that heavily worsen the HRQoL of PwMS in almost all domains of life (Benedict et al., 2005). Furthermore, some comprehensive assessments indicated that these psychological aspects of the disease play a greater role in determining the HRQoL of PwMS than physical state and other (clinical, social, and demographic) characteristics (Benedict et al., 2005). While the merit of the above‐mentioned studies is unquestionable, the independent contribution of various cognitive dysfunction and clinical disability to the quality of life is only rarely investigated.

Thus, the aims of our study were to:

1. Explore the effect of the sociodemographic and clinical aspects (age, education, sex, and disease duration), as well as the psychological aspects (fatigue, depression, and CI) of MS on the patients' HRQoL.
2. To determine the most important contributor(s) to MS patients' HRQoL.
3. To further explore the potential differences between the sexes, considering that there are known differences between man and women regarding prognosis and symptoms.

## 2 MATERIALS AND METHODS

### 2.1 Participants and data collection

We have involved a total of 322 patients in the present study. The patients were recruited from the MS outpatient clinic of the Department of Neurology of the University of Szeged, the Jahn Ferenc Dél‐Pest Hospital of Budapest, and the Markhot Ferenc Teaching Hospital of Eger. The study was approved by the Ethics Committee of the Faculty of Medicine, University of Szeged (207/2015‐SZTE). All participants gave their written informed consent in accordance with the Declaration of Helsinki.

Inclusion criteria were (a) informed consent, (b) a definitive MS diagnosis according to the revised McDonald criteria (2011), (c) relapsing–remitting (RRMS—not active, treated patients) or clinically isolated syndrome (CIS—not active, not treated) disease course, (d) age 18 years or older, (e) native language was Hungarian, (f) the patients were in remission and did not receive steroid therapy for at least 30 days previous to the evaluation, (g) an Expanded Disability Status Scale (EDSS) score between 0 and 6.5.

Exclusion criteria were: (a) any substance use, which may interfere with cognitive abilities or psychometric testing, (b) history of chronic alcoholism, (c) any concomitant or previous physical or psychiatric disorder other than MS that could affect the results of cognitive and/or psychiatric testing, or could affect the patients' mood with the exception of depression, (d) any neurological disorder other than MS that could influence the result of the administered psychometric tests, (e) primary‐progressive (PPMS) or secondary progressive (SPMS) disease course.

The sociodemographic and clinical data of the enrolled patients were acquired from the multiple sclerosis registry of the Department of Neurology of the University of Szeged if available (Bencsik et al., 2017). For patients who were not yet in the database or were recruited from one of the other MS outpatient clinics, the data were obtained from the patient documentation delivered by the patient's attending physician.

### 2.2 Physical disability

Physical disability was measured by the EDSS score (Kurtzke, 1983). In each case, the score was determined by the patient's respective neurologist specialist at the time of enrollment.

### 2.3 Neuropsychiatric tests administered

#### 2.3.1 Cognitive impairment

All 322 patients have been evaluated with the validated Hungarian version of the Brief International Cognitive Assessment for MS (BICAMS) test, which is a rapidly administered screening tool with high sensitivity and specificity for the detection of cognitive impairment in MS patients (Sandi et al., 2015). It consists of three subtests, namely the orally administered version of the Symbol Digit Modalities Test (SDMT), the first 5 immediate recall trial of the California Verbal Learning Test‐II (CVLT‐II), and the first 3 immediate recall trial of the Brief Visuospatial Memory Test—Revised (BVMT‐R; Langdon et al., 2012). We considered a patient cognitively impaired if he/she had abnormal scores on at least one of the three tests administered, a criterion proposed by Dusankova et al. in 2012 (Dusankova, Kalincik, Havrdova, & Benedict, 2012). A score on a given test was considered abnormal if it fell outside of the predefined normal values of said test.

#### 2.3.2 Mood

The patients' mood was evaluated by the Beck Depression Inventory II (BDI‐II) questionnaire validated for Hungarian (Beck, Steer, & Brown, 1996). A patient was considered having depressive symptoms if scored at least 13 points on the test, which is in accordance with the standard cutoff point for this test (Beck et al., 1996).

#### 2.3.3 Fatigue

Fatigue was assessed by the use of the Hungarian version of the Fatigue Impact Scale (FIS) questionnaire (Losonczi et al., 2011). It consists of 40 items, each of which is scored from 0 (indicating no problem) to 4 (indicating a severe problem), providing a final scoring scale from 0 to 160. The three internal subscales of the survey examine the physical, the social, and the cognitive aspects of fatigue separately. Scoring higher means fatigue is more significantly affecting a patient's life. For example, someone with a score of 80 is affected by fatigue more than someone with a score of 20. As there is no well‐established cutoff score for this test, we considered a patient to have been burdened by fatigue if one scored at least 40 points on the FIS questionnaire.

#### 2.3.4 Health‐Related Quality of Life

Of the many tools available to measure HRQoL in MS, we used the MSQoL‐54 questionnaire's Hungarian validated version (Fuvesi et al., 2008). This tool was specifically designed for patients suffering from MS by expanding the SF‐36 survey. The questionnaire consists of 36 general questions and 18 MS specific ones, in total conceiving a 54‐item instrument that contains 12 internal subscales, two single‐item measures along with two additional summary scores. We used the MSQoL‐54 survey because it is a very detailed questionnaire; its basis, the SF‐36 survey is widely used in healthy individuals as well, its results are independent of the assessed population and the expanded questionnaire is very sensitive to problems presenting in patients with MS.

### 2.4 Statistical analysis

The most common approach to identify the impact of certain variables on an independent variable is the various forms of multivariate regressions. However, this approach might be misleading especially in those circumstances when predictors are not independent. This is especially true for the to be examined clinical and cognitive disability. Hence, in order to determine a given variable's impact on the evaluated subscale of HRQoL, we utilized the model‐free, partial least squares regression (PLS) analysis. PLS successively extracts latent (Toth et al., 2017) variables from the dependent variables and the predictors in such a way that covariance between the factors and loadings is maximized. With this approach, PLS reduces the dimensionality of the data by providing a weighted linear combination of *X* variables to form orthogonal components that predict the dependent variable. In our analysis, the dependent variables were the subscales of the MSQoL‐54 questionnaire and the preditors were the scores describing the clinical and cognitive disability. The statistical inference on the significance of the latent variable was carried out by permutation tests on the singular values of the decomposition (5,000 permutations). We regarded the parameter to have an impact on the given subscale if the variable importance of projection (VIP) score was ≥1 (Wold, Johansson, & Cocchi, 1994). The greater the score, the more important a given variable is to the model. Variables with VIP scores <1 are less important and are usually good candidates to be omitted from the model. To evaluate any differences regarding clinical and sociodemographic variables, we used one‐way ANOVA and Fisher's exact test.

## 3 RESULTS

In the present study, a total of 322 patients, 102 (31.6%) men, and 220 (68.4%) women were involved. Of the participants enrolled, 151 (46.9%) spent 12 or fewer years studying, and 171 (53.1%) had been educated for more than 12 years. The mean age of our cohort was 43 ± 11.90 (Range: 21–69) years. Mean disease duration was 12.5 ± 8.0 (Range: 1–45) years. The average EDSS score of the patients was 1.95 ± 1.60 (Range: 0–6.5) points. Cognitive impairment was present in 164 (50.9%) of our subjects. The prevalence of fatigue was 52.2% (168 patients), while the prevalence of depression was 27.0% (87 patients; Table 1).

**Table 1:** The sociodemographic characteristics of our cohort

Sociodemographic data of the cohort	
Age at enrollment (min, max, *SD*) [years]	42.96 (21, 69, 11.88)
EDSS (min, max, *SD*) [points]	1.95 (0, 6.5, 1.57)
Disease duration (min, max, *SD*) [years]	12.48 (1, 45, 7.95)
No. of patients with high education (%)	171 (53.1)
Patients with fatigue (%)	168 (52.2)
Patients with depression (%)	87 (27.0)
Patients with cognitive impairment (%)	164 (50.9)
No. of patients enrolled	322

Note

All of the variables' values are shown in mean.

Abbreviations: EDSS, Expanded Disability Status Scale Score; max, maximum; min, minimum; *SD*, standard deviation.

Regarding the comparison of the sexes, we only found a significant difference in the rate of CI; it was present in 63.7% of the men (65 patients), while 45.0% of women (99 patients; *p* < .002; Table 2).

**Table 2:** Comparison of the clinical and sociodemographic data of the different sexes

	Men	Women	Difference (*p* value)	*F* value
Mean age at enrollment (min, max, *SD*) [years]	41.34 (22.0, 68.0, 12.10)	43.71 (21.0, 69.0, 11.72)	.096	2.781
Mean EDSS score (min, max, *SD*) [points]	2.16 (0.0, 6.5, 1.73)	1.85 (0.0, 6.5, 1.48)	.097	0.000
Disease duration ( min, max, *SD*) [years]	12.48 (1.0, 45.0, 8.74)	12.48 (1.0, 41.0, 7.57)	.997	2.768
No. of patient with high education (%)	56 (54.9)	115 (52.3)	.661	Not applicable
Patients with fatigue (%)	50 (49.0)	118 (53.6)	.473	Not applicable
Patients with depression (%)	25 (24.5)	62 (28.2)	.590	Not applicable
Patients with cognitive impairment (%)	65 (63.7)	99 (45.0)	.002	Not applicable
No. of patients	102	220	Not applicable	Not applicable

Note

All of the variables' values are shown in mean.

Abbreviations: EDSS, Expanded Disability Status Scale Score; max, maximum; min, minimum; *SD*, standard deviation.

We evaluated all of the examined attributes' impact on all of the 14 subscales separately, both for the whole cohort and for men and women independently. Regarding the whole population, depression and fatigue were found to have the most ubiquitous and robust effect on any given subdivision of the HRQoL composite.

Of all the examined variables, only depression and fatigue in general were the sole aspects to have a clinically meaningful (VIP score > 1), negative influence on all the 14 measured subscales of a patient's perceived HRQoL. Fatigue in general had the strongest influence in all of the subscales assessed; however, all of its subsets (cognitive, physical, and social fatigue were included, every other type of fatigue had been excluded) had a significant impact as well on most of the subscales, with the most prominent being physical fatigue, followed by cognitive, and social fatigue. Physical fatigue had a major negative impact on all 14 subscales when evaluating the whole cohort as well as when stratified by sexes. Social and cognitive fatigue had a significant negative influence on all subscales when assessing the whole cohort; however, when subgrouped by sexes they did not reach the threshold in a few subscales. Social fatigue did not reach the threshold (VIP score < 1) in the physical health domain for men, and in the sexual function for women. Cognitive fatigue fell short of being a significant predictor for the change in health, emotional well‐being, overall quality of life, satisfaction with sexual function, and social function domains for men, yet it remained a significant influencing factor on all subscales for women.

Age, education, and EDSS had an impact only on 3, 1, and 2 subscales, respectively, while disease duration and CI were not found to be a meaningful predictor in any of the subscales. Age had a negative effect on the sexual function, satisfaction with sexual function, and physical health dimensions. Having high education made a statistically significant effect on only one, the social function subscale. Patients with more than 12 years of education evaluated their social life to be negatively affected by the disease. A higher EDSS score has been found to negatively influence participation restriction and role limitation among persons with MS in the physical domain. In the emotional domain, the patient's physical health had no impact, only fatigue and depression had an impact on one's role limitation due to emotional problems. With regard to the variables affecting a patient's emotional well‐being, fatigue and depression were found to be the only ones with significant effect. In all other domains where both fatigue and depression had a significant effect, the two of them had a similar share of the cumulative impact. In this case, however, fatigue's impact was marginal, only depression had an effect as powerful as seen in the other subscales. With respect to the pain, energy, and health perception dimensions, similarly to the majority of the other subscales, only depression and fatigue had a significant effect.

When the sexes were compared, of all the aspects examined, depression and fatigue were the only variables to have a major impact on all of the 14 examined domains of HRQoL (VIP > 1) for both men and women.

Yet, on 10 of the domains, differences were found between the sexes. In case of men, in addition to fatigue and depression, at least one other determinant was found to have a negative effect on the HRQoL in 9 domains, while in case of the women such was found only in two domains. Cognitive impairment for men was a negative contributor on the overall quality of life, role limitation due to emotional problems, sexual functions, and the satisfaction with sexual functions domain. In the case of male patients, a higher EDSS score had negatively affected the health perceptions dimension of self‐reported HRQoL. For both sexes, the EDSS score was a negative determinant for the physical health (weaker contributor for women than for men) and physical role limitations subscales. Age was an additional clinical predictor for the change in health, physical health dimensions for men and for the sexual functions, and satisfaction with sexual functions subscale for women. Men who had studied for at least 13 years felt that their HRQoL was also affected by a decline in their social function (Table 3).

**Table 3:** Variable importance in projection (VIP) scores of the different factors contributing to the different subscales of MSQoL‐54 in the whole cohort and in the different sexes

	Sex	Education	Age	Disease duration	EDSS	BVMT‐R score	SDMT score	CVLT‐II score	Physical fatigue	Social fatigue	Cognitive fatigue	Total fatigue	Depression
Change in health	Men	0.606	1.412	0.657	0.869	0.393	0.116	0.117	1.240	1.294	0.942	1.304	1.572
	Women	0.475	0.747	0.375	0.885	0.385	0.515	0.265	1.486	1.236	1.521	1.550	1.164
	Overall	0.530	0.943	0.466	0.915	0.414	0.438	0.242	1.507	1.320	1.452	1.575	1.321
Cognitive function	Men	0.624	0.822	0.477	0.623	0.309	0.488	0.795	1.442	1.165	1.420	1.494	1.291
	Women	0.432	0.674	0.532	0.646	0.663	0.765	0.653	1.332	1.369	1.342	1.475	1.257
	Overall	0.512	0.756	0.534	0.650	0.562	0.665	0.719	1.430	1.378	1.431	1.553	1.330
Emotional well‐being	Men	0.205	0.540	0.452	0.451	0.588	0.807	0.752	1.246	1.477	0.962	1.376	1.773
	Women	0.529	0.698	0.537	0.615	0.516	0.422	0.644	1.349	1.105	1.314	1.382	1.741
	Overall	0.453	0.681	0.531	0.579	0.550	0.546	0.708	1.384	1.271	1.269	1.449	1.836
Energy	Men	0.139	0.651	0.546	0.569	0.437	0.416	0.445	1.434	1.416	1.328	1.550	1.465
	Women	0.379	0.667	0.416	0.702	0.187	0.309	0.145	1.457	1.260	1.613	1.570	1.348
	Overall	0.321	0.693	0.478	0.677	0.263	0.337	0.248	1.517	1.365	1.598	1.636	1.446
Health distress	Men	0.247	0.547	0.286	0.787	0.369	0.445	0.327	1.566	1.361	1.136	1.536	1.595
	Women	0.332	0.460	0.275	0.719	0.488	0.348	0.604	1.483	1.198	1.417	1.506	1.565
	Overall	0.321	0.494	0.287	0.767	0.485	0.399	0.553	1.569	1.296	1.398	1.580	1.637
Health perception	Men	0.418	0.858	0.579	1.128	0.373	0.336	0.179	1.463	1.212	1.094	1.424	1.538
	Women	0.470	0.856	0.436	0.820	0.357	0.432	0.277	1.454	1.146	1.500	1.498	1.368
	Overall	0.469	0.870	0.502	0.976	0.413	0.465	0.269	1.510	1.215	1.429	1.533	1.467
Overall quality of life	Men	0.333	0.411	0.519	0.711	1.093	1.207	0.799	1.237	1.015	0.866	1.183	1.716
	Women	0.551	0.487	0.108	0.844	0.458	0.522	0.575	1.359	1.194	1.347	1.427	1.673
	Overall	0.518	0.472	0.228	0.850	0.665	0.752	0.669	1.395	1.217	1.284	1.439	1.761
Pain	Men	0.195	1.029	0.928	0.996	0.189	0.172	0.213	1.327	1.282	1.265	1.435	1.370
	Women	0.522	0.791	0.471	0.686	0.233	0.422	0.186	1.411	1.246	1.439	1.495	1.515
	Overall	0.439	0.915	0.659	0.814	0.204	0.312	0.197	1.450	1.313	1.450	1.545	1.538
Physical health	Men	0.226	1.163	0.882	1.491	0.628	0.368	0.216	1.427	0.876	1.198	1.321	1.018
	Women	0.438	0.964	0.690	1.149	0.368	0.492	0.315	1.429	1.059	1.485	1.451	1.063
	Overall	0.396	1.066	0.778	1.294	0.448	0.451	0.296	1.496	1.059	1.470	1.484	1.100
Role limitation due to emotional problems	Men	0.513	0.946	0.600	0.820	0.482	0.471	0.988	1.307	1.191	1.162	1.364	1.429
	Women	0.308	0.574	0.441	0.787	0.632	0.680	0.347	1.320	1.321	1.365	1.459	1.516
	Overall	0.398	0.740	0.526	0.832	0.593	0.605	0.597	1.380	1.342	1.363	1.498	1.562
Role limitation due to physical problems	Men	0.401	1.043	0.841	1.305	0.246	0.230	0.203	1.409	1.082	1.287	1.410	1.178
	Women	0.375	0.820	0.569	1.013	0.273	0.501	0.252	1.413	1.135	1.521	1.480	1.288
	Overall	0.398	0.913	0.669	1.128	0.273	0.428	0.248	1.478	1.176	1.525	1.532	1.316
Sexual function	Men	0.077	0.894	0.598	0.648	0.889	0.434	0.724	1.311	1.460	1.204	1.472	1.204
	Women	0.198	1.189	0.625	0.817	0.166	0.471	0.160	1.509	0.987	1.321	1.418	1.486
	Overall	0.183	1.183	0.638	0.757	0.307	0.381	0.309	1.546	1.161	1.367	1.514	1.495
Satisfaction with sexual function	Men	0.040	0.993	0.791	0.885	1.086	0.519	0.949	1.170	1.327	0.863	1.259	1.340
	Women	0.322	1.213	0.579	0.775	0.038	0.288	0.165	1.531	1.064	1.307	1.451	1.445
	Overall	0.258	1.217	0.683	0.850	0.292	0.365	0.422	1.527	1.210	1.258	1.491	1.506
Social function	Men	0.541^a^	0.772	0.934	1.059	0.642	0.685	0.439	1.223	1.522	0.932	1.372	1.217
	Women	0.190	0.345	0.470	0.765	0.106	0.177	0.159	1.620	1.176	1.335	1.540	1.676
	Overall	0.348	0.515	0.704	0.967	0.220	0.448	0.297	1.553	1.370	1.246	1.556	1.588

Note

All VIP scores from all the latent factors examined by the PLS analysis were in concordance with each other; therefore, all the values shown in the table are that of latent variable no. 1, except for education's influence on men's social function, where latent factor no. 1 fell out of line from all other concordant and significant VIP scores for the rest latent factors.

a VIP scores latent factors 1–5 are as follows: 0.541, 1.402, 1.386, 1.382, 1.381.

## 4 DISCUSSION

In our study, we evaluated the possible predicting factors of HRQoL in a large, homogenous population of MS patients. Our model‐free analysis indicated that the most significant contributor to quality of life is depression and fatigue. Besides these symptoms, specific subscales of MSQ54 were driven—to a much lesser extent—primarily by age, EDSS, and education. Furthermore, for men and women, different contributors were observed to influence the HRQoL, and with different level of influence.

Assessing and analyzing the HRQoL of PwMS is a demanding task, as it is a complex—and by nature—a subjective concept. Most tools used for its evaluation are based on self‐report, therefore the results, despite being quantified measures, often remain a question to interpretation. Furthermore, significant deviation can be seen in results in the literature when psychopathologic symptoms are examined because of the different tools (with different length, depth or primary focus) used. Additionally, assessing the HRQoL of a patient only at a given point of time or once during a long(er) follow‐up period is not satisfactory, because possible determinants can change over time. Despite these issues, focusing on and addressing a possible decline the subjective QoL of the patients—in particular regard to the medically alterable and measurable aspects—should be a highly important medical goal.

Among the many possible and most often investigated predictors of quality of life, we examined clinical disability, cognitive dysfunction, fatigue, and depression. While EDSS is not a perfect measure of disability, in the range what we have examined (EDSS: 0–6.5 points), it is nearly linear and correlates well with the patient's physical state. For the evaluation of cognitive impairment, we used the BICAMS test, an easy and rapidly administrable test in the everyday hospital setting, which is being validated to swiftly increasing amount of languages, possibly becoming the gold standard screening tool to assess a patient's cognitive capabilities, meanwhile covering the most commonly affected cognitive domains in PwMS (Corfield and Langdon, 2018). Fatigue and depression were assessed by one of the most ubiquitous screening tools in use the FIS and BDI questionnaires, respectively.

The prevalence of depression (27.0%), fatigue (52.2%), CI (50.9%) in our cohort was in line the literature (Ben Ari Shevil et al., 2014; Chiaravalloti & DeLuca, 2008). With more than 300 patients assessed, the sample size made it possible to stratify between sexes, we confirmed our previous and other results regarding the difference of CI's prevalence between men and women (Patti et al., 2015; Sandi et al., 2017).

Regarding the combined results of men and women, our results are in agreement with recent studies to a great extent; however, some differences are present. Depression and fatigue are by far the leading predictors of perceived HRQoL in all domains (Benedict et al., 2005; Fuvesi et al., 2010). Other factors were restricted to one or two domains only; EDSS to the physical aspects, educational status to the social domain. Surprisingly, CI—in general—appears to be a minor predictor. Current results on this topic are controversial. Many studies showed cognitive impairment to be a primary determinant of patients' HRQoL, on the other hand, some studies relying on self‐reported HRQoL measures did not find CI to be a significant factor (Miller & Allen, 2010; Mitchell, Benito‐Leon, Gonzalez, & Rivera‐Navarro, 2005). The reason behind the inconsistency of these results is multifactorial. One possible reason is that CI affects the vocational status, thus these patients are unable to report their problems sufficiently (Benedict et al., 2005). Another reason might be that patients with serious CI many times are unaware of their deficit, sometimes even affected by euphoria and moria, which make their self‐reports unrealistic (Benedict, Priore, Miller, Munschauer, & Jacobs, 2001; Benedict et al., 2004). As was concluded by Benedict et al., CI seems to be mainly predictive of what PwMS are actually capable of doing, rather what they are able to report (Benedict et al., 2005). Yet another reason for cognitive impairment's minor relationship with our cohort's HRQoL might be the patients' cognitive reserve capacity. Higher levels of active and passive cognitive reserve capacity have been shown to be associated with lower levels of perceived disability, higher levels of functional health, and higher levels of well‐being in patient‐reported outcome surveys (Schwartz, Snook, Quaranto, Benedict, & Vollmer, 2013).

As for the gender‐specific impact of MS on the patients' HRQoL, the literature is scarce. Men were found to perceive their HRQoL significantly worse than women especially in the physical and social functioning and vitality domains (Casetta et al., 2009). It was also shown that male patients face a worse prognosis in general compared with women (Greer & McCombe, 2011; Zaffaroni & Ghezzi, 2000). In addition, prospective studies have found that a worse HRQoL can be a significant predictor for the change in the patients' disability status (Nortvedt, Riise, Myhr, & Nyland, 2000).

None of these studies have investigated whether there is a difference between the factors influencing HRQoL in different sexes though. In the current literature, information about gender's effect on the HRQoL is scarce, ours is among the first studies to show a difference between the sexes regarding the factors that influence HRQoL in PwMS. As for men, their physical status and their age—which are two naturally intertwining aspects of one's physical decline over the years, hastened by MS—had a more profound and powerful impact on more subscales than it did for women. An explanation for this could be how western societies still attribute great importance to the role of physical disability, and how a person's “value” with a visible physical disability is perceived. Despite the rapid advancement of IT technology, many jobs have emerged that require no classical physical endurance and vitality, our culture still places great value upon appearances, how a person's stature emanates efficiency and capability. Living with a visible physical disability could induce a sensation of failure to meet social standards and to cease to be the provider in a household, which could be a more serious problem for men than women (Barnwell & Kavanagh, 1997; Duval, 1984).

We hypothesize that the reason cognitive impairment solely influenced the HRQoL of men but not women is due to the fact that CI's prevalence is much higher in men, and thus its impact has reached a threshold where its burden becomes a significant factor (Sandi et al., 2017). The presence of CI makes an MS patient more likely to get divorced, thus self‐reportedly he feels a significant decline in his sexual life (Hakim et al., 2000). Also as discussed before, a person with CI is not able to pinpoint exactly the problem present (and thus generally has a better overall HRQoL questionnaire), but to an extent feels that something has changed and, as we demonstrated CI can have a negative impact on one's overall quality of life, without influencing the different, more exact subscales individually. Having spent more time in the educational system predicted worse scores on the social HRQoL domain for men, which was expected, as it has been shown that people with higher education have better prospects at well‐paying jobs, better work hours, and economic prosperity (Weintraub et al., 2011). From this higher social state, a sudden fall—may it be real or only perceived—due to a debilitating sickness can decrease the HRQoL of a PwMS to a great extent, especially for men who are still the wage‐earners in many societies.

## 5 CONCLUSION

The concept of HRQoL is far more complex than simply the physical impact as most care models would traditionally relate. Our assessment further underlines this opinion, as we have shown that the less evaluated, yet frequently prevalent psychological burdens of the disease (particularly depression and fatigue) are the main determinants of the self‐reported well‐being of PwMS. We have confirmed other studies' findings (Yalachkov et al., 2019), that fatigue and depression are the main determinants of MS patients' HRQoL not only at disease onset, as previously shown (Nourbakhsh, Julian, & Waubant, 2016; when one would expect a patient to have a drop in their HRQoL, and become burdened with depression, after being diagnosed with a lifelong, degenerative disease), but years after the diagnosis as well. Therefore the evaluation and treatment of PwMS should be conducted on a much broader spectrum than regular physical and MRI examinations, even in the youngest population (Rainone et al., 2017), a pre‐emptive approach is proposed. These findings invoke the need for the regular assessment of depression, fatigue, cognition, and other psychopathological aspects with a multidimensional, quantitative approach.

Also, ours is one of the first studies in the literature to show that different burdens influence the HRQoL of men and women, and with different strength. This further supports the need for the complex and personalized care for these patients, furthermore, regular psychopathological assessments and periodic feedback regarding a patient's HRQoL are urged especially since different determinants influence the HRQoL of men and women. It also proves the need for involvement of psychological and psychiatric specialists and teamwork for the proper management of patients burdened by MS, as many of these symptoms can be ameliorated and managed by not only a psychopharmacological, but by a psychotherapeutic approach as well.

## 6 STUDY LIMITATIONS

One of the limitations of this study was the lack of assessment of patient's anxiety, which has been shown to be a major contributing factor to the HRQoL of PwMS (Janssens et al., 2003; Salehpoor, Rezaei, & Hosseininezhad, 2014). Another limitation is the absence of administration of detailed neuropsychological tests and evaluation of the active and passive cognitive reserve capacity of our patients, which was beyond the scope and primary focus of this article, but is nonetheless a crucial element in the long‐term treatment of MS patients living with cognitive impairment. The strong points of our paper include the relatively big cohort, the wide range of variables assessed and the strong statistical model measuring every examined variable's impact on the HRQoL of our patients and novel data on the difference of predictors constituting the HRQoL of men and women.

## CONFLICT OF INTEREST

The authors report no conflict of interest.

## Data Availability

The data that support the findings of this study are available from the corresponding author upon reasonable request.
